# Radar Imaging of Non-Uniformly Rotating Targets via a Novel Approach for Multi-Component AM-FM Signal Parameter Estimation

**DOI:** 10.3390/s150306905

**Published:** 2015-03-23

**Authors:** Yong Wang

**Affiliations:** Research Institute of Electronic Engineering Technology, Harbin Institute of Technology, Harbin 150001, China; E-Mail: wangyong6012@hit.edu.cn; Tel.: +86-451-8641-8051

**Keywords:** radar imaging, modified version of Chirplet decomposition, IHAF

## Abstract

A novel radar imaging approach for non-uniformly rotating targets is proposed in this study. It is assumed that the maneuverability of the non-cooperative target is severe, and the received signal in a range cell can be modeled as multi-component amplitude-modulated and frequency-modulated (AM-FM) signals after motion compensation. Then, the modified version of Chirplet decomposition (MCD) based on the integrated high order ambiguity function (IHAF) is presented for the parameter estimation of AM-FM signals, and the corresponding high quality instantaneous ISAR images can be obtained from the estimated parameters. Compared with the MCD algorithm based on the generalized cubic phase function (GCPF) in the authors’ previous paper, the novel algorithm presented in this paper is more accurate and efficient, and the results with simulated and real data demonstrate the superiority of the proposed method.

## 1. Introduction

Radar imaging of non-uniformly rotating targets has developed for about two decades [1,2,3,4,5,6]. It is assumed that the target is engaged in complex maneuvers, and the classical Range-Doppler (RD) algorithm is ineffective to generate a well-focused radar image because of the time varying character for the Doppler frequency of each scatterer contribution [7]. Then, the Range-Instantaneous-Doppler (RID) technique was proposed to deal with this problem. For the RID algorithm, the azimuth focusing is implemented by the time frequency analysis for the non-stationary signal in a certain range cell. The performance of non-stationary signal has been studied for a long time, and many useful results have been obtained [8,9].

One kind of algorithms for time frequency analysis is based on the high resolution time frequency distribution (TFD) with reduced cross-terms, such as the smoothed-pseudo-Wigner-Ville distribution (SPWVD) [10], or the high order TFD [11,12,13], but these algorithms still suffer from the tradeoff between the time frequency resolution and cross-terms. The other kind of algorithms for time frequency analysis is based on parametric techniques. These algorithms model the received signal in a range cell as multi-component amplitude-modulated and frequency-modulated (AM-FM) signals after motion compensation. By estimating the parameters of each component, the focused radar images can be obtained with the RID technique. Considering the complications of AM-FM signal parameter estimation, some approximated signal models have been investigated recently. In [3,14], the received signal in a range cell is modeled as a multi-component linear frequency modulated (LFM) signal with constant amplitude, and some efficient algorithms have been proposed, but these algorithms are only valid in the situation where the target’s maneuverability is not too severe. For targets with significantly complex motion, high order phase terms will exist in the azimuth echoes. Then, the received signal in a range cell can be modeled as a multi-component cubic phase signal. Some efficient algorithms for the parameters estimation of cubic phase signal are proposed in [15,16,17,18], and the radar image quality can be improved compared with the LFM signal model. 

For the AM-FM signal model, an efficient algorithm for the parameters estimation of it is the Chirplet decomposition. By decomposing the AM-FM signal into parametric, redundant well localized components, its energy curve in the time-frequency plane can be approximated as the combination of a set of beelines. Some Chirplet decomposition algorithms are proposed in [19,20,21,22], and most of them have been used in the field of radar imaging successfully. The Chirplet atom has the form of LFM signal model with Gaussian envelop, and it is inappropriate to characterize the complicated time varying performance for the instantaneous frequency. Then, the modified version of Chirplet atom and polynomial Chirplet transform are proposed in [23,24,25,26], where the Chirplet atom is extended to the form of polynomial phase signal. But the corresponding signal decomposition algorithms for them are complicated and thus suffer from the computational load. 

In this paper, the modified version of Chirplet atom is used to characterize the AM-FM signal, and a novel signal decomposition algorithm based on integrated high order ambiguity function (IHAF) is proposed. This algorithm requires only one-dimensional (1D) maximizations to estimate the third order coefficient for the modified version of Chirplet atom, and the other parameters can be obtained by the Dechirp technique and Fourier transform. The novel algorithm is used in radar imaging of maneuvering target, and the high quality instantaneous radar images can be obtained consequently. 

This paper is organized as follows: in Section 2, the multi-component AM-FM signal model for the azimuth received signal is established. In Section 3, the principle and implementation of modified version of Chirplet decomposition based on IHAF are proposed. The corresponding radar imaging algorithm for the non-uniformly rotating target is presented in Section 4. Section 5 is the radar imaging results for simulated and real data. Section 6 is the conclusion for the paper.

## 2. Signal Model

**Figure 1:**
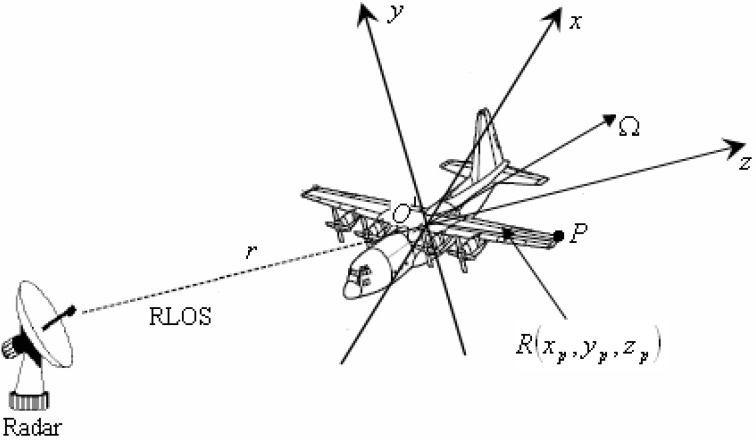
Radar imaging geometry of target with non-uniform rotation.

A plane target with complex motion is used here as an example to define the radar imaging geometry, as shown in Figure 1. It is assumed that the motion compensation has been implemented, and the target can be considered as a “turntable” target with rotating center *O* in the (*x*, *y*, *z*) Cartesian coordinate. It is assumed that the unit vector of the radar line of sight (RLOS) is *r*, and the *z*-axis is determined by it. Ω is the synthetic vector for the angular velocity of the rotating target, and the *x*-axis is determined by the *z*-axis and Ω. That is to say, the synthetic vector Ω is located in the *x* − *z* plane. Then, the *y*-axis is determined by the *z*-axis and *x*-axis as *y* = *z* × *x*, where × denotes outer product. A random scatterer *P* on the target is selected, and it is represented by the vector *R*(*x_p_*, *y_p_*, *z_p_*) from the rotating center *O* to the position of point *P*.

The purpose of radar imaging is to obtain a focused two-dimensional radar image on the image projection plane. The high range resolution is determined by the large bandwidth for the transmitted signal, and the high cross-range resolution is obtained by the relative rotation between the radar and target. Then, the motion compensation should be implemented before the radar imaging procedure. The motion compensation includes the range alignment and phase adjustment.

The purpose of range alignment is to compensate the translational component of each scatterer after range compressing, and the purpose of phase adjustment is to remove the Doppler phase caused by the translation. After motion compensation, the imaging target can be considered as a turn table target rotating around a reference point. In this case, the received signal in a range cell can be modeled as multi-component amplitude-modulated and frequency-modulated (AM-FM) signals, which can be illustrated as follows:

*Step 1*: The Doppler frequency for scatterer P can be expressed as:

(1) $$f_{d}=\frac{2\left[\Omega •\left(R\times r\right)\right]}{\lambda }$$

where • denotes the inner product, λ represents the wavelength.

*Step 2*: For the time varying character of angular velocity of the rotating target, the synthetic vector Ω can be expressed as:

(2) $$\Omega =\sum_{m=0}^{K}\alpha_{m}t^{m}$$

where $\alpha_{0}$ is the constant term, $\alpha_{m}\left(m=1,2,\ldots ,K\right)$ are coefficients of the first, second and high order terms of Ω. *t* is the azimuth time, and *K* is the phase order.

*Step 3*: Substitute Equation (2) into Equation (1), the Doppler frequency can be further written as:

(3) $$f_{d}=\frac{2}{\lambda }\left[\left(\sum_{m=0}^{K}\alpha_{m}t^{m}\right)•\left(R\times r\right)\right]$$

*Step 4*: The distance from scatterer *P* to radar can be computed as:

(4) $$Dis\left(t\right)=-\frac{\lambda }{2}\int_{t_{0}}^{t}f_{d}dt=-\int_{t_{0}}^{t}\left[\sum_{m=0}^{K}\alpha_{m}•\left(R\times r\right)t^{m}\right]dt=R_{0}-\sum_{m=1}^{K+1}\frac{\alpha_{m-1}•\left(R\times r\right)t^{m}}{m}$$

where $R_{0}$ denotes the initial distance from radar to the target center at the initial time $t_{0}$ .

*Step 5*: Assume that there are *Q* scatterers in a range cell, and the received signal can be modeled as multi-component AM-FM signals as follows:

(5) $$\begin{array}{l} s\left(t\right)=\sum_{i=1}^{Q}A_{i}\left(t\right)\text{exp}\left(-j\frac{4\pi }{\lambda }Dis_{i}\left(t\right)\right)\\ =\sum_{i=1}^{Q}A_{i}\left(t\right)\text{exp}\left[j\frac{4\pi }{\lambda }\left(-R_{0}+\sum_{m=1}^{K+1}\frac{\alpha_{m-1}•\left(R_{i}\times r\right)t^{m}}{m}\right)\right] \end{array}$$

where $A_{i}\left(t\right),i=1,2,\ldots ,Q$ is the time varying amplitude of the *i* th component.

From the steps above, we can see that for the maneuvering target, the received signal in a range cell can be modeled as multi-component AM-FM signals after motion compensation. In order to obtain the focused two-dimensional radar images in this case, the parameters for the multi-component AM-FM signals should be estimated with high precision. Hence, the modified version of Chirplet decomposition based on integrated high order ambiguity function (IHAF) is presented for the parameters estimation of AM-FM signals in this paper, and the corresponding radar imaging scheme is presented simultaneously. Then, the radar image quality can be improved combined with the RID technique.

## 3. Modified Version of Chirplet Decomposition Based on IHAF

### 3.1. Principle of Modified Version of Chirplet Decomposition

**Figure 2:**
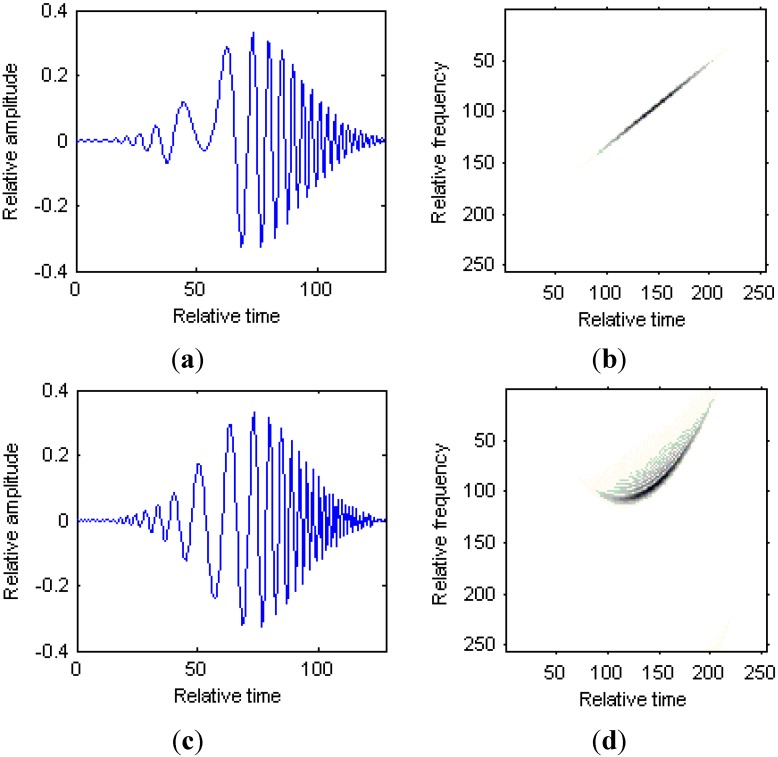
Comparison between Chirplet atom and modified version of Chirplet atom. (**a**) Time series for the Chirplet atom; (**b**) WVD for the Chirplet atom; (**c**) Time series for the modified version of Chirplet atom; (**d**) WVD for the modified version of Chirplet atom.

The modified version of Chirplet atom is defined in [23] as follows:

(6) $$g_{k}\left(t\right)=\sqrt[{4}]{\frac{1}{\pi \sigma_{k}^{2}}}\text{exp}\left\{-\frac{{\left(t-t_{k}\right)}^{2}}{2\sigma_{k}^{2}}+j\omega_{k}\left(t-t_{k}\right)+j\beta_{k}{\left(t-t_{k}\right)}^{2}+j\gamma_{k}{\left(t-t_{k}\right)}^{3}\right\}$$

where the parameter $\left(t_{k},\omega_{k}\right)\in R$ determines the time and frequency center, $\left(\beta_{k},\gamma_{k}\right)\in R$ denotes the chirp rate and curvature, and the variance $\sigma_{k}\in R^{+}$ controls the width for the modified version of Chirplet atom. Figure 2 shows the comparison between the traditional Chirplet atom and modified version of Chirplet atom. Figure 2a is the time series for the Chirplet atom; Figure 2b is the Wigner-Ville distribution (WVD) for the Chirplet atom; Figure 2c is the time series for the modified version of Chirplet atom; Figure 2d is the WVD for the modified version of Chirplet atom.

We can see from Figure 2 that the curvature for the modified version of Chirplet atom has a bending effect on the traditional Chirplet atom, and thus it is suitable for the analysis of signals with strongly nonlinear instantaneous frequencies. 

Then, for an arbitrary analytic signal s(t) , it can be expressed by the sum of $g_{k}\left(t\right)$ as follows:

(7) $$s\left(t\right)=\sum_{k=0}^{\infty }C_{k}g_{k}\left(t\right)$$

where $C_{k}$ is the weighted coefficient to be estimated.

Then, the basic principle of modified version of Chirplet decomposition can be illustrated as follows:

*Step 1*: Design a modified version of Chirplet atom $g_{0}\left(t\right)$ with the condition that the distance between s(t) and its orthogonal projection on $g_{0}\left(t\right)$ is minimum, which is equivalent to:

(8) $${\left|C_{0}\right|}^{2}=\underset{g_{0}}{\text{max}}{\left|\langle s_{0}\left(t\right),g_{0}\left(t\right)\rangle \right|}^{2},s_{0}\left(t\right)=s\left(t\right)$$

*Step 2*: Compute the remainder signal $s_{1}\left(t\right)$ after $g_{0}\left(t\right)$ is obtained, just as follows:

(9) $$s_{1}\left(t\right)=s_{0}\left(t\right)-C_{0}g_{0}\left(t\right)$$

*Step 3*: Repeat the steps above until the residual energy satisfies a given threshold, we obtain:

(10) $$s_{k}\left(t\right)=s_{k-1}\left(t\right)-C_{k-1}g_{k-1}\left(t\right)$$

It is obvious from Equation (8) that the estimation of $C_{k}$ requires multi-dimensional maximizations, which suffers from high computational load. In this paper, a novel modified version of Chirplet decomposition algorithm is proposed, which is based on the integrated high order ambiguity function (IHAF) proposed in [27]. The novel modified version of Chirplet decomposition algorithm requires only one-dimensional maximizations with high precision, and it will be illustrated in the next section.

### 3.2. Modified Version of Chirplet Decomposition Based on IHAF

The IHAF algorithm is based on the high order ambiguity function (HAF) proposed in [27]. The HAF can estimate the parameters of a cubic phase signal. But for multi-component signals, the cross-terms will appear and the auto-terms can not be detected correctly. Hence, the IHAF is proposed to reduce the cross-terms between different components. In this section, the curvature $\gamma_{k}$ for the modified version of Chirplet atom is estimated by the IHAF algorithm, and then the other parameters are estimated by the Dechirp technique and Fourier transform.

For a weighted modified version of Chirplet atom with the discrete form:

(11) $$s\left(t\right)=D_{k}\sqrt[{4}]{\frac{1}{\pi \sigma_{k}^{2}}}\text{exp}\{-\frac{{\left(t-t_{k}\right)}^{2}}{2\sigma_{k}^{2}}+j\omega_{k}\left(t-t_{k}\right)+j\beta_{k}{\left(t-t_{k}\right)}^{2}+j\gamma_{k}{\left(t-t_{k}\right)}^{3}\}$$

The high order ambiguity function (HAF) is defined in [27] as follows:

(12) $$S\left(\Theta ,m_{1},m_{2}\right)=\int_{-\infty }^{+\infty }s\left(t+m_{1}+m_{2}\right)s\left(t-m_{1}-m_{2}\right)s^{*}\left(t+m_{1}-m_{2}\right)s^{*}\left(t-m_{1}+m_{2}\right)\text{exp}\left(-j\Theta m_{1}m_{2}t\right)dt$$

where $m_{1}$ and $m_{2}$ are lags different from zero, ${\left(\cdot \right)}^{*}$ denotes the conjugate.

Substituting Equation (11) into Equation (12), we obtain:

(13) $$\begin{array}{l} S\left(\Theta ,m_{1},m_{2}\right)=D_{k}^{4}{\left(\pi \sigma_{k}^{2}\right)}^{-1}\text{exp}\left[-\frac{2\left(m_{1}^{2}+m_{2}^{2}\right)}{\sigma_{k}^{2}}+8jm_{1}m_{2}\beta_{k}\right]\times \\ \int_{-\infty }^{+\infty }\text{exp}\left(-\frac{2{\left(t-t_{k}\right)}^{2}}{\sigma_{k}^{2}}\right)\text{exp}\left[24j\gamma_{k}m_{1}m_{2}\left(t-t_{k}\right)\right]\text{exp}\left[-jm_{1}m_{2}\Theta t\right]dt \end{array}$$

Let $t-t_{k}=t^{\prime }$ , we can rewrite Equation (13) as follows:

(14) $$\begin{array}{l} S\left(\Theta ,m_{1},m_{2}\right)=D_{k}^{4}{\left(\pi \sigma_{k}^{2}\right)}^{-1}\text{exp}\left[-\frac{2\left(m_{1}^{2}+m_{2}^{2}\right)}{\sigma_{k}^{2}}+8jm_{1}m_{2}\beta_{k}\right]\text{exp}\left[-jm_{1}m_{2}\Theta t_{k}\right]\times \\ \int_{-\infty }^{+\infty }\text{exp}\left(-\frac{2{t^{\prime }}^{2}}{\sigma_{k}^{2}}\right)\text{exp}\left[24j\gamma_{k}m_{1}m_{2}t^{\prime }\right]\text{exp}\left[-jm_{1}m_{2}\Theta t^{\prime }\right]dt^{\prime }\\ =D_{k}^{4}{\left(\pi \sigma_{k}^{2}\right)}^{-1}\text{exp}\left[-\frac{2\left(m_{1}^{2}+m_{2}^{2}\right)}{\sigma_{k}^{2}}+8jm_{1}m_{2}\beta_{k}\right]\text{exp}\left[-jm_{1}m_{2}\Theta t_{k}\right]\times \\ \int_{-\infty }^{+\infty }\text{exp}\left(-\frac{2t^{2}}{\sigma_{k}^{2}}\right)\text{exp}\left[-jm_{1}m_{2}t\left(\Theta -24\gamma_{k}\right)\right]dt \end{array}$$

By using the following formula:

(15) $$\int_{-\infty }^{+\infty }\text{exp}\left(-At^{2}\pm 2Bt+C\right)dt=\sqrt{\frac{\pi }{A}}\text{exp}\left(\frac{B^{2}}{A}+C\right)$$

where A≠0 and Re(A)≥0 .

We obtain:

(16) $$\left|S\left(\Theta ,m_{1},m_{2}\right)\right|=D_{k}^{4}{\left(\sqrt{2\pi }\sigma_{k}\right)}^{-1}\text{exp}\left[-\frac{2\left(m_{1}^{2}+m_{2}^{2}\right)}{\sigma_{k}^{2}}\right]\text{exp}\left[-\frac{1}{8}\sigma_{k}^{2}m_{1}^{2}m_{2}^{2}{\left(\Theta -24\gamma_{k}\right)}^{2}\right]$$

Thus, the $\left|S\left(\Theta ,m_{1},m_{2}\right)\right|$ yields a peak at $\Theta =24\gamma_{k}$ , and the curvature *γ_k_* for the modified version of Chirplet atom can be readily obtained as:

(17) $$\gamma_{k}=\text{arg}\underset{\Omega }{\text{max}}\left|S\left(\Theta ,m_{1},m_{2}\right)\right|>/24$$

From the definition of HAF in Equation (12), we can see that the HAF has the nonlinearity character. Hence, the cross-terms will appear for multi-component modified version of Chirplet atoms. Then, we can use the IHAF algorithm to reduce the cross-terms, and the auto-terms can be amplified by the integration operation simultaneously. The IHAF is defined as follows:

(18) $$G\left(\Theta \right)=\int_{-\infty }^{+\infty }\int_{-\infty }^{+\infty }S\left(\Theta ,m_{1},m_{2}\right)dm_{1}dm_{2}$$

So, for multi-component modified version of Chirplet atoms, the curvature *γ_k_* for the signal component with maximum energy should be estimated as follows:

(19) $$\gamma_{k}=\text{arg}\underset{\Omega }{\text{max}}\left|G\left(\Theta \right)\right|>/24$$

After the curvature *γ_k_* is estimated, the other parameters can be estimated by the Dechirp technique and Fourier transform. The parameters for other modified version of Chirplet components can be estimated combined with the CLEAN technique [28].

### 3.3. Numerical Example

A two components modified version of Chirplet atoms is considered in this section to demonstrate the performance of HAF and IHAF algorithms for the signal decomposition procedure. The parameters for the two components modified version of Chirplet atoms with discrete form are shown in Table 1, where it is assumed that the sampling rate is unity, and t∈[−127,127] .

**Table 1:** Parameters of the simulated signal.

Components (*k*)	*D_k_*	*σ_k_*	*t_k_*	*ω_k_*	*β_k_*	*γ_k_*
1	4	40	18	0.4	5×10^−3^	1×10^−5^
2	4	60	50	0.8	−5×10^−3^	−2×10^−5^

**Figure 3:**
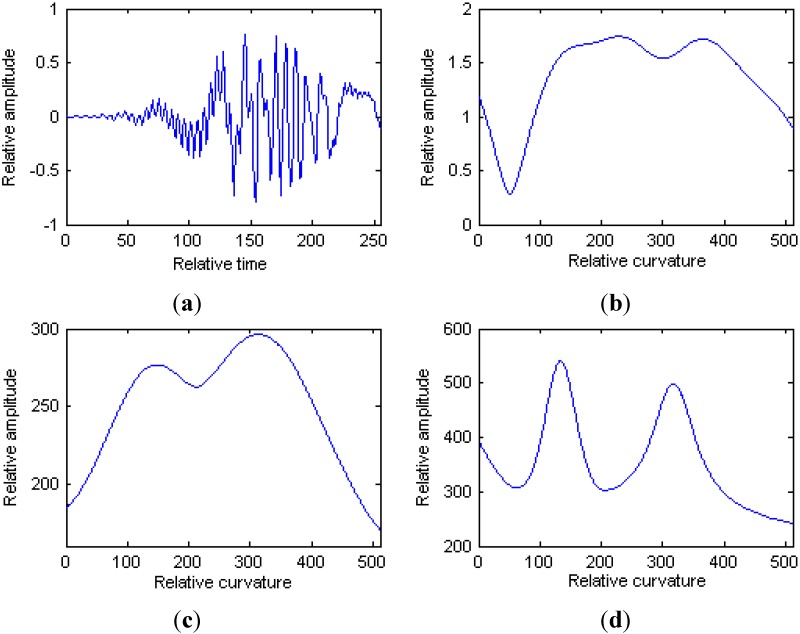
Results of the numerical example. (**a**) Simulated signal; (**b**) HAF for the signal; (**c**) IHAF with lags $m_{1}\in [1:10]$ and $m_{2}\in [11:20]$ ; (**d**) IHAF with lags $m_{1}\in [1:20]$ and $m_{2}\in [21:40]$ .

The simulated signal in time domain is shown in Figure 3a. Figure 3b is the HAF for the signal, and the modified version of Chirplet atoms can not be detected for the nonlinearity character of HAF. Figure 3c is the IHAF for the signal, where the lags $m_{1}$ and $m_{2}$ are selected as $m_{1}\in [1:10]$ and $m_{2}\in [11:20]$ . We can see that there exist two peaks in the IHAF for the signal, and the modified version of Chirplet atoms can be detected by the peak positions. Figure 3d is the IHAF for the signal with lags $m_{1}\in [1:20]$ and $m_{2}\in [21:40]$ . It is obvious that with the increase of lag numbers in IHAF, the resolution of curvature estimation is improve simultaneously.

## 4. Radar Imaging Based on Modified Version of Chirplet Decomposition

For radar imaging of non-uniformly rotating target, the received signal in a range cell can be modeled as multi-component AM-FM signals after motion compensation, which has been illustrated in Section 2. Then, the modified version of Chirplet decomposition based on IHAF algorithm is proposed in Section 3 to analyze the multi-component AM-FM signals. In this section, the corresponding novel radar imaging algorithm is presented as follows:

*Step 1*: Assume that the received signal in a range cell is Q components AM-FM signals, as shown in Equation (5).

*Step 2*: Approximate the multi-component AM-FM signals in a range cell as a weighted sum of N components modified version of Chirplet atoms of the form:

(20) $$s\left(t\right)=\sum_{k=0}^{N-1}C_{k}g_{k}\left(t\right)$$

where $g_{k}\left(t\right)$ has the form of Equation (6).

*Step 3*: Initialize k=0 , and $s_{0}\left(t\right)=s\left(t\right)$ .

*Step 4*: Estimate $\gamma_{k}$ for $s_{k}\left(t\right)$ by finding the peak of G(Θ) as Equation (19).

*Step 5*: Dechirp the k th modified version of Chirplet component $s_{k}\left(t\right)$ to the form of traditional Chirplet atom by constructing the reference signal $s_{ref1}\left(t\right)=\text{exp}\left(-j\gamma_{k}t^{3}\right)$ . We obtain:

(21) $$\begin{array}{l} s_{dechirp}\left(t\right)=s_{k}\left(t\right)\cdot s_{ref1}\left(t\right)=C_{k}{\left(\pi \sigma_{k}^{2}\right)}^{-0.25}\text{exp}\left[-\frac{{\left(t-t_{k}\right)}^{2}}{2\sigma_{k}^{2}}\right]\text{exp}\left[j\left(\beta_{k}-3\gamma_{k}t_{k}\right)t^{2}\right]\\ \times \text{exp}\left[j\left(\omega_{k}-2\beta_{k}t_{k}+3\gamma_{k}t_{k}^{2}\right)t\right]\text{exp}\left[j\left(-\omega_{k}t_{k}+\beta_{k}t_{k}^{2}-\gamma_{k}t_{k}^{3}\right)\right] \end{array}$$

*Step 6*: For the traditional Chirplet atom $s_{dechirp}\left(t\right)$ , the chirp rate ${\hat{\beta }}_{k}=\beta_{k}-3\gamma_{k}t_{k}$ can be estimated by the existing methods, such as the integrated cubic phase function (ICPF) algorithm proposed in [29]. Then, the Chirplet atom can be further dechirped to a sinusoidal signal, and the time center $t_{k}$ can be estimated by the Wigner-Ville distribution (WVD) for the sinusoidal signal with the peak position. The detailed implementation of it can be found in [30].

*Step 7*: The chirp rate for the first modified version of Chirplet atom can be estimated as:

(22) $$\beta_{k}={\hat{\beta }}_{k}+3\gamma_{k}t_{k}$$

*Step 8*: The other parameters, including $\omega_{k}$ , $\sigma_{k}$ and $C_{k}$ can be estimated by the Fourier transform and the similar algorithm as in [30];

*Step 9*: Subtract the estimated k th component from $s_{k}\left(t\right)$ based on CLEAN technique;

*Step 10*: Set k=k+1 , and repeat the above steps until k=N−1 or the residual signal energy is less than a threshold.

**Figure 4:**
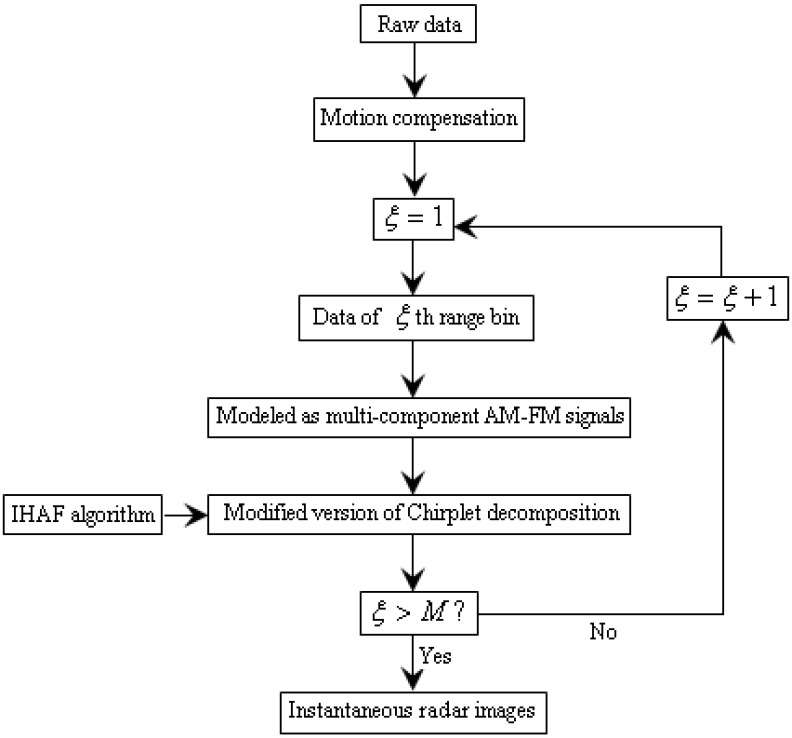
Flowchart of radar imaging algorithm in this paper.

Then, the instantaneous radar images can be obtained by the procedure above combined with the RID technique. Figure 4 is the flowchart for the radar imaging algorithm proposed in this paper, where M is the number of range bins.

## 5. Radar Imaging Results

In this section, the radar imaging results for simulated and real data are provided to demonstrate the effectiveness of the IHAF algorithm for modified version of Chirplet decomposition for radar imaging of maneuvering target.

### 5.1. Simulated Data

**Figure 5:**
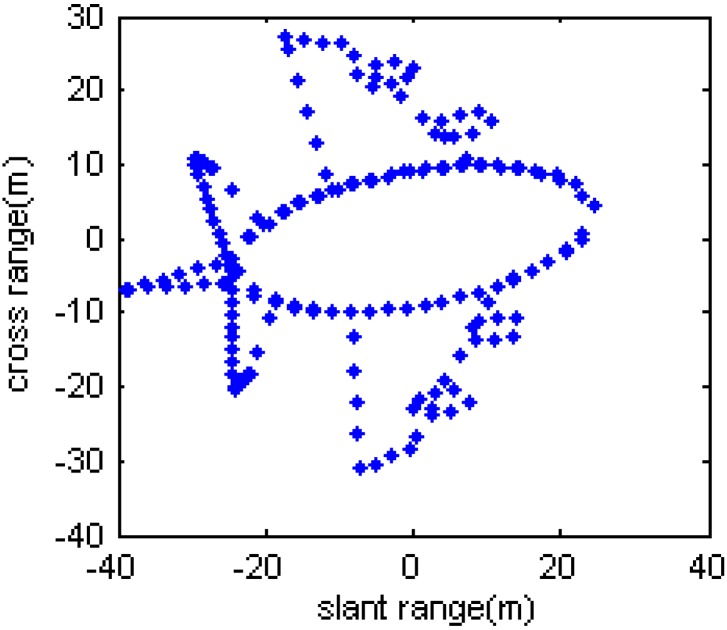
Simulated target model.

The parameters for the simulated data are shown as follows: the carrier frequency for the transmitted LFM signal is *f_0_* = 5.52 GHz, the bandwidth is *B* = 400 Hz, the pulse width is 25.6 µs. After motion compensation, it is assumed that the target is rotating with equal changing acceleration, and the rotating parameters are as follows: the initial velocity is 0.021 rad/s, the acceleration is 0.015 rad/s^2^, and the acceleration rate is 1.6 rad/s^3^. Figure 5 shows the simulated target model, and it consists of 193 scatterers. 

**Figure 6:**
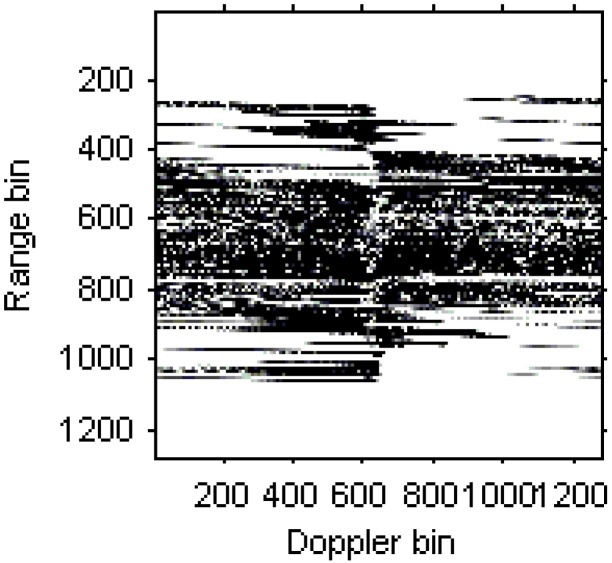
Radar image based on the RD algorithm.

Figure 6 is the radar image based on the RD algorithm. It is obvious that the image has been blurred severely for the high maneuverability of the target.

**Figure 7:**
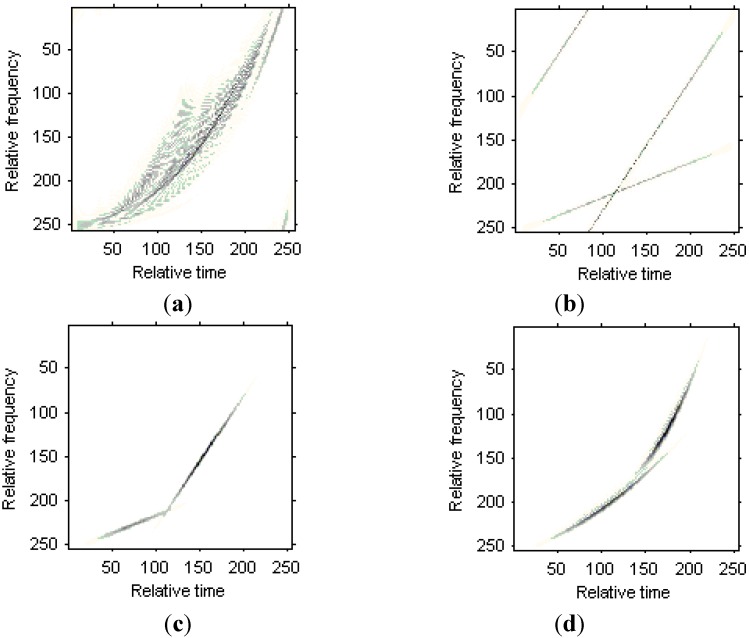
Time frequency representations for the received signal in a range bin. (**a**) WVD for the original signal; (**b**) WVD for two LFM signal components; (**c**) WVD for two Chirplet components; (**d**) WVD for two modified version of Chirplet components.

The WVD for the received signal in the 50th range bin is shown in Figure 7a, and the nonlinear character for the Doppler frequency is obvious. Figure 7b is the WVD for the two LFM signal components estimated from the original signal, Figure 7c is the WVD for the two Chirplet components estimated from the original signals, and Figure 7d is the WVD for the two modified version of Chirplet components estimated from the original signals. We can see that the modified version of Chirplet decomposition algorithm has better performance in the presentation for the original signal.

**Figure 8:**
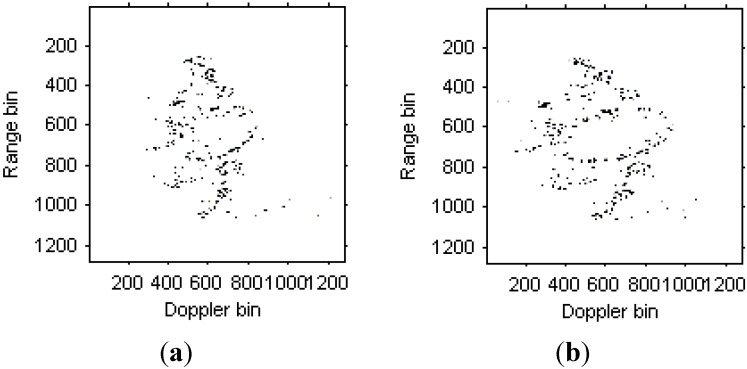
Radar images based on LFM signal model. (**a**) Radar image at time *t* = 0.17 s; (**b**) Radar image at time *t* = 0.22 s.

**Figure 9:**
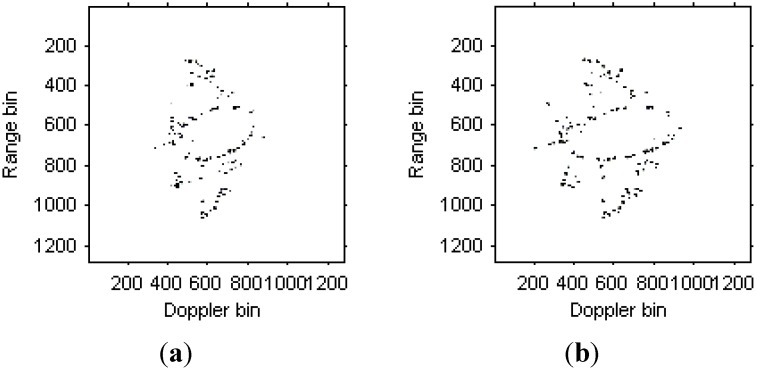
Radar image based on Chirplet decomposition algorithm. (**a**) Radar image at time *t* = 0.17 s; (**b**) Radar image at time *t* = 0.22 s.

The instantaneous radar images at different time positions based on the LFM signal model are shown in Figure 8. It can be seen that the images quality has been improved greatly compared with Figure 6. Then, the instantaneous radar images at the same time positions as Figure 8 by the traditional Chirplet decomposition algorithm are shown in Figure 9. 

**Figure 10:**
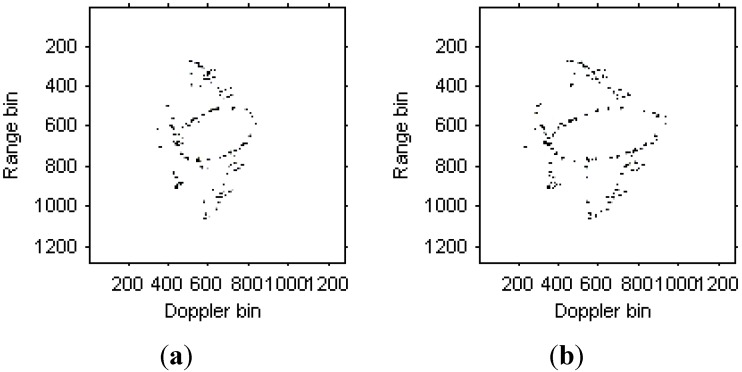
Radar image based on modified version of Chirplet decomposition algorithm proposed in [31]. (**a**) Radar image at time *t* = 0.17 s; (**b**) Radar image at time *t* = 0.22 s.

**Figure 11:**
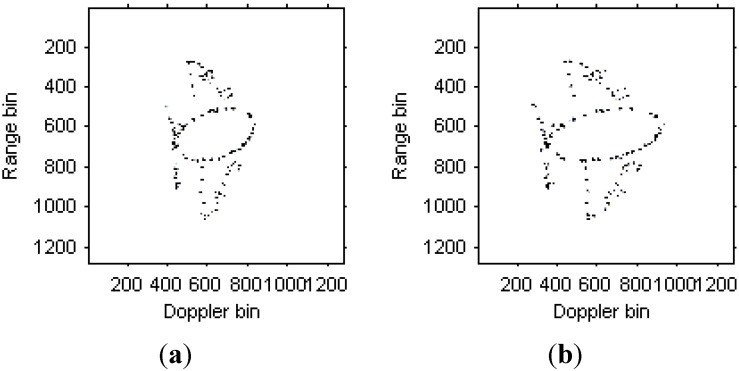
Radar image based on modified version of Chirplet decomposition algorithm proposed in this paper. (**a**) Radar image at time *t* = 0.17 s; (**b**) Radar image at time *t* = 0.22 s.

We can see that that images quality has been further improved. Figure 10 are the instantaneous radar images at the same time positions as Figure 8 and Figure 9 based on the modified version of Chirplet decomposition algorithm proposed in [31], and the instantaneous radar images based on the novel modified version of Chirplet decomposition algorithm proposed in this paper are shown in Figure 11.

Comparing Figure 8, Figure 9 and Figure 10, it is obvious that the image quality in Figure 11 is better, especially in the part of the wings of the target. This demonstrates that the images quality for the modified version of Chirplet decomposition algorithm proposed in this paper is better than the traditional radar imaging algorithms. Here, we give a quantitative comparison of the radar images in Figure 8, Figure 9, Figure 10 and Figure 11 by the entropy criterion with the conclusion that better focused image has smaller entropy [32]. The entropy is computed by the definition in [32] (where the entropy is calculated without the normalization procedure for the image, this is equivalent to the original definition and thus it is a negative number), and the corresponding results are shown in Table 2 as follows:

**Table 2:** Entropies of radar images in Figure 8, Figure 9, Figure 10 and Figure 11.

Figures	(a)	(b)
Figure 8	−3.5625×10^6^	−3.3216×10^6^
Figure 9	−7.0477×10^6^	−6.3434×10^6^
Figure 10	−8.3932×10^6^	−6.4800×10^6^
Figure 11	−9.3333×10^6^	−6.8215×10^6^

It is obvious from Table 2 that, the entropies for the radar images in Figure 11 are smaller than those in Figure 8, Figure 9 and Figure 10. This also demonstrates the superiority of the modified version of Chirplet decomposition algorithm in this paper.

### 5.2. Real Data

**Figure 12:**
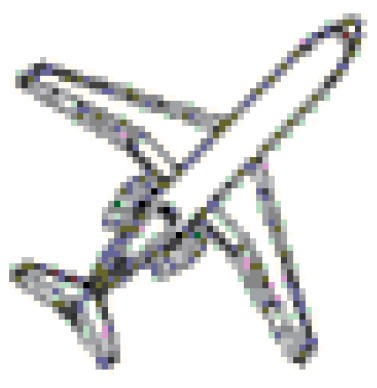
Optical picture of the plane.

For the real data, the radar parameters are the same with the simulated data. The raw data is collected by the radar receiver for the Yak-42 plane with the length of 36.38 m, the width of 34.88 m and the height of 9.83 m. An optical picture of the target is shown in Figure 12.

**Figure 13:**
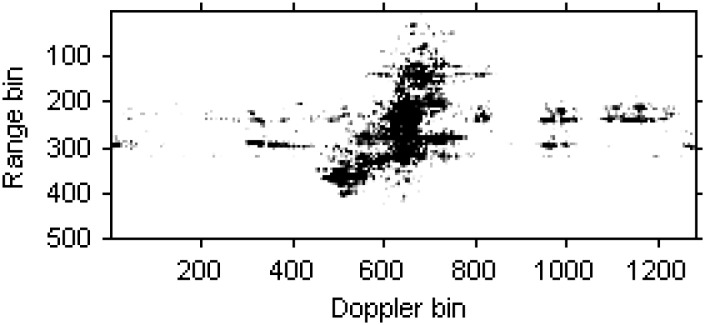
Radar image based on the RD algorithm.

The blurred radar image based on RD algorithm is shown in Figure 13.

**Figure 14:**
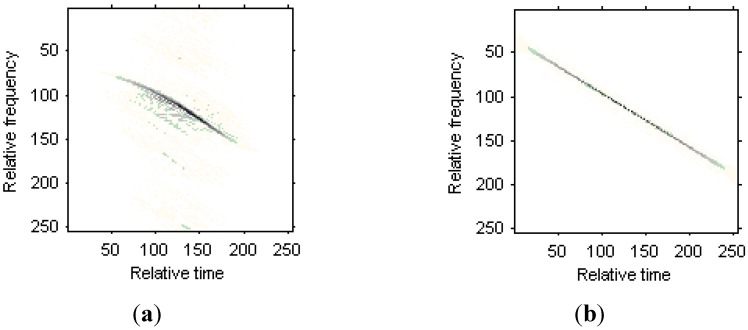

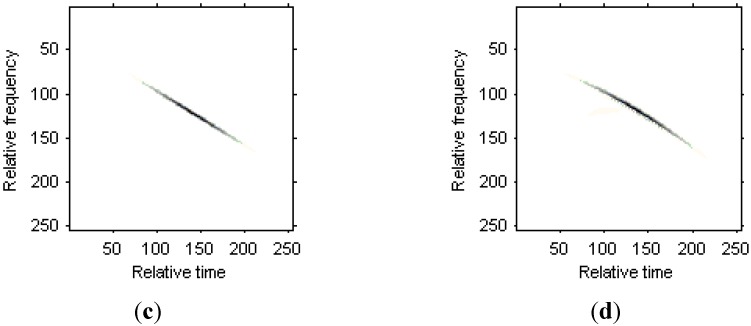
Time frequency representations for the received signal in a range bin. (**a**) WVD for the original signal; (**b**) WVD for the LFM signal component; (**c**) WVD for the Chirplet component; (**d**) WVD for the modified version of Chirplet component.

The WVD for the received signal in the 35th range bin is shown in Figure 14a. Figure 14b is the WVD for the LFM signal model estimated from the original signal, Figure 14c is the WVD for the Chirplet component estimated from the original signals, and Figure 14d is the WVD for the modified version of Chirplet component estimated from the original signals. We can see that the modified version of Chirplet decomposition algorithm has better performance in the presentation for the original signal.

**Figure 15:**
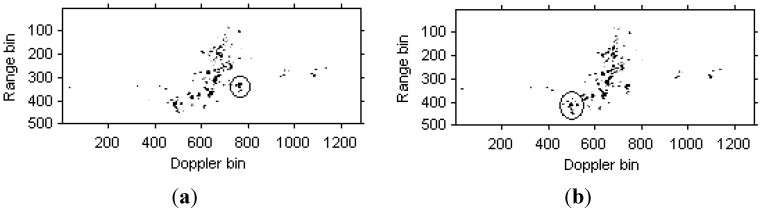
Radar images based on LFM signal model. (**a**) Radar image at time *t* = 1.01 s; (**b**) Radar image at time *t* = 1.23 s.

**Figure 16:**
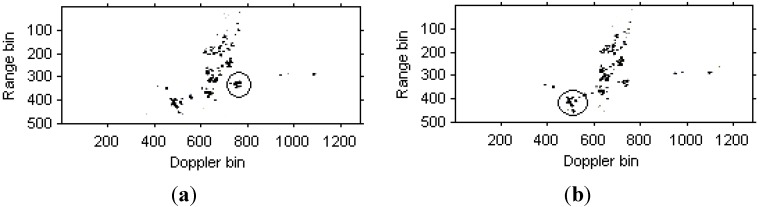
Radar images based on Chirplet decomposition algorithm. (**a**) Radar image at time *t* = 1.01 s; (**b**) Radar image at time *t* = 1.23 s.

Figure 15a,b are the instantaneous radar images at different time positions based on the LFM signal model. Figure 16a,b are the radar images at the same time positions as Figure 15 based on the traditional Chirplet decomposition algorithm. It is obvious that the images quality has been improved compared with Figure 13.

**Figure 17:**
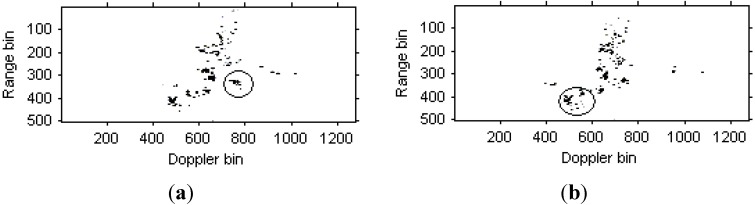
Radar image based on modified version of Chirplet decomposition algorithm proposed in [31]. (**a**) Radar image at time *t* = 1.01 s; (**b**) Radar image at time *t* = 1.23 s.

**Figure 18:**
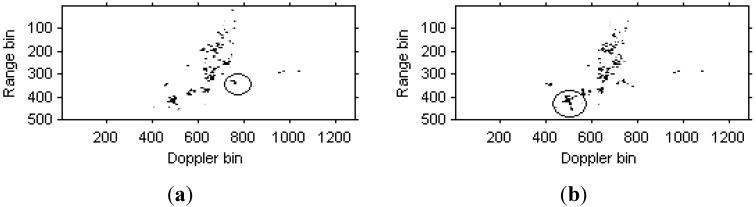
Radar images based on modified version of Chirplet decomposition algorithm proposed in this paper. (**a**) Radar image at time *t* = 1.01 s; (**b**) Radar image at time *t* = 1.23 s.

Figure 17a,b are the instantaneous radar images at the same time positions as Figure 15 and Figure 16 based on the modified version of Chirplet decomposition algorithm proposed in [31], and the instantaneous radar images based on the novel modified version of Chirplet decomposition algorithm are shown in Figure 18a,b, respectively.

It can be seen from Figure 15, Figure 16, Figure 17and Figure 18 that the focus performance of the modified version of Chirplet decomposition algorithm in this paper is better than other algorithms, especially in the elliptical parts of the ISAR images.

**Table 3:** Entropies of radar images in Figure 15, Figure 16, Figure 17and Figure 18.

Figures	(a)	(b)
Figure 15	−5.9369×10^7^	−5.9417×10^7^
Figure 16	−6.5060×10^7^	−1.5350×10^8^
Figure 17	−7.3117×10^7^	−1.6493×10^8^
Figure 18	−7.8240×10^7^	−1.7073×10^8^

The entropies for the radar images in Figure 15, Figure 16, Figure 17 and Figure 18 are shown in Table 3. This demonstrates the superiority of the novel algorithm in this paper.

## 6. Conclusions

For radar imaging of maneuvering targets, the received signal in a range cell can be modeled as multi-component AM-FM signals. In this paper, the modified version of Chirplet decomposition based on the IHAF algorithm is proposed to analyze the AM-FM signals. This algorithm decomposes the AM-FM signals into the combination of a series of modified version of Chirplet atoms, and when combined with the RID technique, high quality instantaneous radar images can be obtained. Results of simulated and real data demonstrate the validity of the novel algorithm proposed in this paper.

## Nomenclature

*O*	Rotating center of the target
*r*	Unit vector of the RLOS
×	Outer product
●	Inner product
*P*	Random scatterer on the target
*λ*	Wavelength
Ω	Synthetic vector for the angular velocity of the rotating target
*K*	Polynomial phase order
*α*_0_	Constant term
*R*_0_	Initial distance from radar to the target center
*t*_0_	Initial time
*Q*	Number of scatterers in a range cell
*t_k_*	Time center of the modified version of Chirplet atom
*ω_k_*	Frequency center of the modified version of Chirplet atom
*β_k_*	Chirp rate of the modified version of Chirplet atom
*γ_k_*	Curvature of the modified version of Chirplet atom
*σ_k_*	Width for the modified version of Chirplet atom
*C_k_*	Weighted coefficient
(⋅)^∗^	Conjugate
*m*_1_	Time lags
*m*_2_	Time lags
